# Influence of body fatness on the functional status in older patients with postherpetic neuralgia

**DOI:** 10.1097/MD.0000000000045269

**Published:** 2025-10-24

**Authors:** Ji-Seon Son, Jeewoon Joung, Aram Doo

**Affiliations:** a Department of Anesthesiology and Pain Medicine, Jeonbuk National University Medical School, Jeonju, South Korea; b Research Institute of Clinical Medicine of Jeonbuk National University-Biomedical Research Institute of Jeonbuk National University Hospital, Jeonju, South Korea.

**Keywords:** body composition, fat, muscle, neuralgia, postherpetic neuralgia, zoster

## Abstract

Although there has been a few studies investigating the impact of increased body mass index on chronic pain disorders, the results are conflicting. In the current study, body composition such as percent body fat (PBF) was quantitatively assessed using bioelectrical impedance analysis. This study aimed to investigate the impact of the extent of body fat on the functional status in older patients with postherpetic neuralgia. Older patients, aged 50 years or above, who was diagnosed with postherpetic neuralgia were enrolled in this study. All participants underwent body composition analysis at their initial visit. Simultaneously, functional status including pain-related interference in activities of daily living (ADL), quality of life (QOL), and the emotional well-being was evaluated by questionnaires. These included the Zoster brief pain inventory, the EuroQoL-5 dimension (EQ-5D), and WHO-5 well-being index. Based on the results of body composition analysis, participants were divided into 2 groups, the normal adiposity (PBF of < 17% for men and < 32% for women) and the high adiposity group (PBF of ≥17% for men and ≥32% for women), and outcome measures were compared between 2 groups. A total of 75 participants (35 in the normal adiposity and 40 in the high adiposity group) were analyzed. Pain intensity evaluated by an 11-point numeric rating scale were comparable between the normal adiposity and the high adiposity group (4.0 [2.0–5.0] vs 4.0 [3.0–6.0], *P = *.374). However, a total score of Zoster brief pain inventory was higher in the high adiposity group, indicating worse performance in ADL, compared to the normal adiposity group (*P = *.041 by 2-tailed *t*-test). And the EQ VAS (0 = the worst health you can imagine, 100 = the best health you can imagine) was 70.7 ± 20.0 and 55.0 ± 23.8 in the normal and the high adiposity groups (*P = *.008). WHO-5 well-being index was comparable between the 2 groups (*P = *.082). The results of the study suggest that individuals with higher body fat may experience reduced function in ADL and decreased QoL compared to those with normal body composition, even when the severity of pain is comparable.

## 1. Introduction

Postherpetic neuralgia (PHN), which is generally defined as persistent pain lasting more than 90 days after skin rash,^[1]^ is the most common and challenging complication of herpes zoster (HZ). The chronic symptoms of PHN is often severe and debilitating, and it can persist for months or years.^[2,3]^ PHN may affect the physical, functional, psychological, and social ability of the patients in their daily life. The more severe pain intensity, the greater interference with daily activities.^[4]^ Several investigations reported that PHN reduced quality of life (QoL) and interfered with the activities of daily living (ADL) of the patients, and consequently may increase socioeconomic burden.^[3,5,6]^ Therefore, while alleviating pain is the primary goal of PHN treatment, it is also important to predict its potential impact on functional status and QoL.

It has been suggested that obesity is associated with the development of chronic pain disorders such as neuropathic pain as well as musculoskeletal pain.^[7–10]^ Kerckhove et al reported that over-weighted or obese patients may be at a higher risk of developing or suffering from chronic pain disorder.^[7]^ Meanwhile, a cross-sectional study for acute HZ patients suggested that increased body mass index (BMI) of 24 kg/m^2^ or more might be related to the lower pain score and the fewer opioid consumption.^[11]^ The authors assumed that these conflicting results may be partly caused by the limitation of the diagnostic criteria for obesity. BMI, which is the weight in kilogram divided by height in meter squared, is not the accurate measurement of adipose tissue. It can reflect neither the extent nor the distribution of body fat.

Along with this concept, body composition such as percent body fat (PBF) was quantitatively evaluated using bioelectrical impedance analysis (BIA) in the current study. To date, few well-designed studies have investigated the association between different body composition characteristics and the severity of pain and functional status. This prospective observational study aimed to investigate the impact of the extent of body fat on the functional status of the older patient who was diagnosed with PHN. The authors hypothesized that increased body fat would affect the ability of the older PHN patients to perform ADL. Additionally, its impact on the QoL and emotional well-being were also evaluated.

## 2. Methods

This prospective observational study was approved by the Institutional Review Board of Jeonbuk National University Hospital (IRB number; CUH 2019-12-015). This manuscript adheres to the applicable STROBE guidelines. After obtaining informed consent from all participants, a total of 80 consecutive patients who visited our pain clinic at a tertiary care hospital for PHN were enrolled in the study from March 2020 to February 2023. Inclusion criteria were as follows: age ≥ 50 years at the time of first outpatient visit with a diagnosis of PHN; clinical PHN symptoms; PHN was defined as persistent zoster-related pain lasting more than 90 days after the appearance of a skin rash; and pain intensity with numeric rating scale of 2 or more. Patients with a difficulty in communication such as literacy problem, language difficulties, or cognitive impairment, resulting in an incapability of completing self-administered questionnaires, were excluded from the study. And participants with metal implants, including cardiac pacemakers and implantable cardioverter defibrillators were also excluded because body composition measurement using BIA was contraindicated in such patients.

All participants enrolled in this study underwent body composition analysis using the BIA method at their initial visit to our department. Whole-body segmented multi-frequency BIA was performed with the Inbody S10® device (Biospace, Seoul, Korea). Inbody S10® utilizes a 4-compartment model to assess body composition, dividing it into body water, protein, fat and mineral masses.^[12,13]^ Although BIA is an indirect measurement of body composition, it is cost-effective, easy to use bedside, and radiation-free. And its accuracy has been validated by several studies.^[13,14]^ According to the manufacturer’s guidelines, patients were instructed to rest in a supine position on a nonconductive table for at least 10 minutes before the measurements. The skin surface was cleansed with an alcohol swab before electrode application. Subsequently, the surface electrodes were attached to the thumb and middle finger of each hand and both ankles, according to the manufacturer’s instructions. The measurements provided various parameters such as body fat mass (kg), PBF (%), skeletal muscle mass (kg), and skeletal muscle index (kg/m^2^) for all participants. Muscle fat ratio (MFR) was calculated by dividing the skeletal muscle mass (kg) by the body fat mass (kg).

Based on the results of body composition analysis, participants were divided into 2 groups; the normal adiposity and the high adiposity group. It is important to note that in this study, group classification was based on PBF rather than BMI, and thus the term “high adiposity” is used instead of “obesity” to more accurately reflect excess body fat. Group classification was determined using a predetermined cutoff value of PBF specific to each sex, established in a previous study in Korean adults which defined cutoff for excessive body fatness.^[15]^ The normal adiposity group included participants with a PBF of < 17% for men and < 32 % for women, while the high adiposity group included participants with a PBF of ≥ 17% for men and ≥ 32 % for women. Outcome measures reflecting the functional status of PHN patients were compared between the 2 groups.

## 3. Evaluation of functional status in older patients with PHN

All participants received a booklet and were asked to complete self-rated questionnaires to assess pain-related interference in ADL, QoL, and emotional well-being. These included the Zoster brief pain inventory (ZBPI), the EuroQoL-5 dimension (EQ-5D), and WHO-5 well-being index. A physician was available to assist participants during their initial consultation if needed.

First, ZBPI is a self-administered questionnaire designed to measure pain intensity and its impact on ADL.^[16]^ Pain intensity was assessed using the item 3 of the ZBPI, asking the patient to indicate the “worst” pain experienced over the previous week on an 11-point Likert scale. Those with a score of <2, indicating mild pain, were excluded from further evaluation. Then, participants were asked to quantify the pain-related interference in their daily life across 7 functional categories (general activity, mood, walking ability, work, relations with others, sleep, and enjoyment of life). Each category was also assessed using an 11-point Likert scale (0 = does not interference, and 10 = interferes completely). Participants completed the questionnaires at their initial visit, detailing how the zoster-related pain had affected their ADL over the previous week. The ZBPI total score was calculated as the sum of scores from the 7 categories, ranging from 0 to 70. A higher score indicates worse performance in ADL.

Second, QoL of the participants were assessed with the EQ-5D questionnaire. The EQ-5D is a standardized instrument developed by the EuroQol group to measure generic health-related QoL.^[17]^ It is widely used in clinical and economic studies of healthcare, as well as in population health surveys. The EQ-5D consists of 5 dimensions that address mobility, self-care, usual activities, pain/discomfort, and anxiety/depression. Responses are categorized into 5 levels: no problem, slight problems, moderate problems, severe problems, extremely severe problems, as defined by the EQ-5D-5L (5-level version of EQ-5D).^[17]^ Additionally, the EQ visual analogue scale (EQ VAS) assesses self-rated health on a vertical scale ranging from 0 to 100. The top endpoint was labeled “The best health you can imagine,” while the bottom endpoint was labeled “The worst health you can imagine.” This scale additionally provided a quantitative measure of the patient’s overall health.

Third, WHO-5 well-being index is a simple and widely used tool for assessing subjective psychological well-being of the patients with chronic pain. This scale is developed by the World Health Organization, it consists of 5 statements related to positive mood, vitality, and general interest. Each of 5 statements is self-rated using a 6-point Likert scale: 0 = at no time, 1 = some of the time, 2 = less than half of the time, 3 = more than half of the time, 4 = most of the time, 5 = all of the time. The total score ranges from 0 to 25, and lower scores indicate worse well-being. A few previous studies had suggested that a score below 13 indicated poor well-being and a high risk of depression.^[18,19]^ In the current study, a score below 13 was categorized into poor well-being.

## 4. Sample size calculation and statistical analysis

In the current study, the sample size was predetermined by t-test sample size calculation using IBM SPSS statistics for windows, version 26 (IBM Corp., Armonk) based on the assumption that the minimum detectable difference of ZBPI total score was 10 between the normal and high adiposity groups. A total of 74 participants were required with a significant level of 0.05 (α = .05) and a power of 80% (β = .20). To allow attrition, the total sample size was enlarged to 80.

All statistical analysis were performed using IBM SPSS Statistics for Windows, version 26. Continuous variables were compared using 2-tailed independent-samples *t*-test after normality test. Categorical variables were compared using Mann–Whitney rank-sum test. Univariate linear regression analysis was performed to verify the relationships between patient demographics and the ZBPI total score. The statistically significant variables proved by univariate analysis were then integrated into a multivariate linear regression model, and regression coefficient (β), coefficient of determination (*R*^2^), and *P* values were calculated for each variable. All descriptive data are expressed as the numbers, numbers (percentages), mean ± standard deviations, or median (interquartile range). A *P* value < .05 was considered statistically significant.

## 5. Results

**Figure 1.**
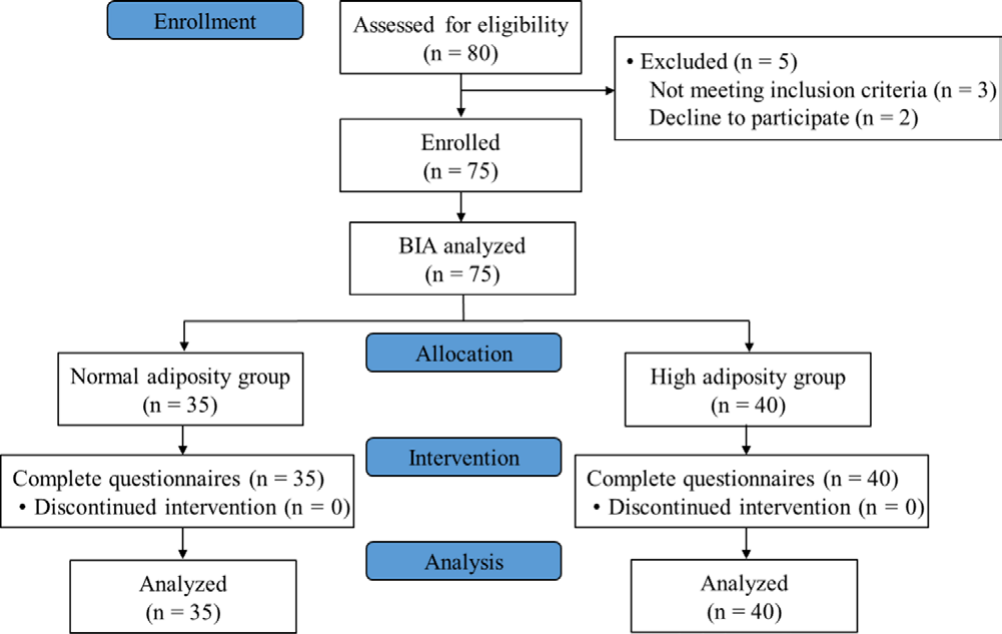
Patient flow chart. BIA = bioelectrical impedance analysis.

Eighty participants were assessed for eligibility, and 75 participants (35 in the normal adiposity and 40 in the high adiposity group) were enrolled for the present study. Patient flow chart is described in Figure 1. The normal adiposity group consisted of 14 men and 21 women, while the high adiposity group included 24 men and 16 women. Patient characteristics were compared between the normal adiposity and the high adiposity groups (Table 1). The average age of the patients in the high adiposity group was higher than in the normal adiposity group (71.0 vs 63.0, *P* = .026). However, no statistically significant differences were observed between the groups for other characteristics, including sex distribution, duration of PHN symptoms, or affected dermatome. Regarding body composition, body fat mass, PBF, and muscle fat ratio were significantly higher in the high adiposity group than in the normal adiposity group for both sexes (*P* < .001).

**Table 1:** Patient demographics and body composition characteristics in the 2 groups.

	Normal adiposity group (n = 35)	High adiposity group (n = 40)	*P*
Age (yr)	63.0 (59.0–70.0)	71.0 (64.0–75.8)	.026*
Female sex [n (%)]	21 (60.0%)	16 (40.0%)	.084
Duration of symptoms (months)	4.0 (1.2–11.2)	5.2 (3.4–35.3)	.177
Affected dermatome (n)			
Thoracic	20	25	.722
Trigeminal	7	7	
Cervical	6	4	
Lumbosacral	2	4	
BMI (kg/m^2^)	22.7 ± 1.9	26.0 ± 3.3	<.001†
Male	22.9 ± 2.3	24.7 ± 2.7	.084
Female	22.6 ± 1.8	27.7 ± 3.2	<.001†
Body fat mass (kg)			
Male	8.6 ± 2.2	16.7 ± 4.6	<.001†
Female	13.8 ± 2.1	24.4 ± 4.5	<.001†
Percent body fat (%)			
Male	12.8 ± 2.7	24.3 ± 5.3	<.001†
Female	24.7 ± 3.5	39.1 ± 4.7	<.001†
Skeletal muscle mass (kg)			
Male	32.9 ± 7.0	28.3 ± 4.4	.121
Female	22.5 ± 3.0	20.0 ± 2.8	.022†
Skeletal muscle index (kg/m^2^)			
Male	9.4 ± 1.2	8.8 ± 1.2	.213
Female	7.7 ± 0.7	7.8 ± 0.8	.748
Muscle fat ratio			
Male	3.9 (3.2–4.2)	1.8 (1.4–2.2)	<.001*
Female	1.7 ± 0.3	0.8 ± 0.2	<.001†

Data are presented as numbers, numbers (percentages) or median (interquartile range).

BMI = body mass index.

* *P* < .05 by Mann–Whitney rank-sum test.

† *P* < .05 by 2-tailed *t*-test.

**Figure 2.**
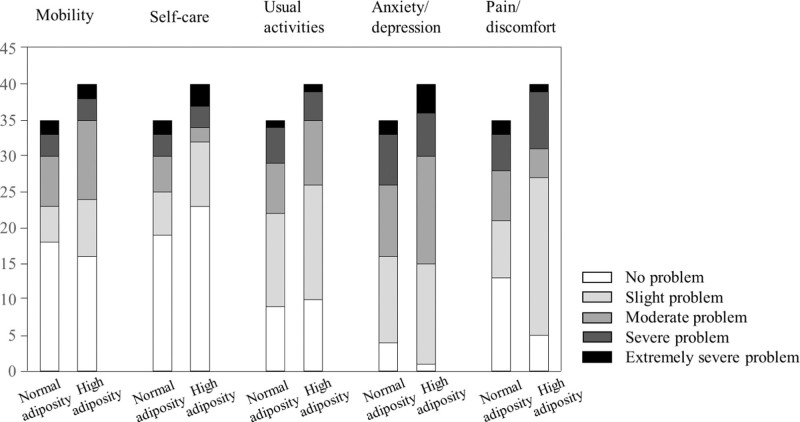
Quality of life evaluated by 5-level version of EuroQoL-5 dimension (EQ-5D-5L) between the normal adiposity and the high adiposity group.

Table 2 presents measures of functional status in PHN patients, including ZBPI, EQ-5D, and WHO-5 well-being index. Pain intensity, evaluated using an 11-point numeric rating scale, was similar between the normal and the high adiposity groups (4.0 [2.0–5.0] vs 4.0 [3.0–6.0], *P* = .374). However, the total ZBPI score was significantly higher in the high adiposity group, indicating worse performance in ADL, compared to the normal adiposity group (*P* = .041 by 2-tailed *t*-test). Notably, significant differences were found in walking ability (*P* = .004) and normal work (*P* = .002), suggesting that higher adiposity may intensify challenges with mobility and work-related tasks, as shown in the subanalysis of the 7 ZBPI subdomains. However, there were no statistically significant differences between groups in general activity, mood, relationships, sleep, or enjoyment of life. The EQ VAS (0 = the worst health you can imagine, 100 = the best health you can imagine) was 70.7 ± 20.0 and 55.0 ± 23.8 in the normal and the high adiposity groups (*P* = .008). Meanwhile, Figure 2 illustrates the quality of life, evaluated by EQ-5D-5L, compared between the normal adiposity and the high adiposity group. Regarding emotional well-being, WHO-5 well-being index and the number of patients with poor well-being were comparable between the 2 groups (*P* = .082).

**Table 2:** Activities of daily living, quality of life and the patients’ well-being in patients with postherpetic neuralgia.

	Normal adiposity group (n = 35)	High adiposity group (n = 40)	*P*
Pain intensity (NRS)	4.0 (2.0–5.0)	4.0 (3.0–6.0)	.374
ZBPI total (0–70)	27.6 ± 15.6	36.6 ± 17.5	.041*
General activity	5.1 ± 3.2	6.0 ± 2.4	.264
Mood	4.9 ± 3.2	6.1 ± 2.5	.129
Walking ability	2.0 (0.0–3.0)	5.0 (1.0–7.0)	.004†
Normal work	2.7 ± 2.2	4.8 ± 3.0	.002*
Relations	3.0 (0.0–5.0)	5.0 (2.0–7.0)	.134
Sleep	5.0 (3.0–8.0)	6.0 (2.0–8.0)	.562
Enjoyment	4.3 ± 3.2	5.9 ± 2.6	.052
EQ-5D-5L (no/slight/moderate/severe/extremely severe problems)			
Mobility	18/5/7/3/2	16/8/11/3/2	.485
Self-care	19/6/5/3/2	23/9/2/3/3	.707
Usual activities	9/13/7/5/1	10/16/9/4/1	.850
Anxiety/depression	4/12/10/7/2	1/14/15/6/4	.347
Pain/discomfort	13/8/7/5/2	5/22/4/8/1	.359
EQ VAS (0–100)	70.7 ± 20.0	55.0 ± 23.8	.008*
WHO-5 well-being index (0–25)	9.8 ± 5.3	10.1 ± 5.5	.082
No. of poor well-being (%)	22 (62.9%)	28 (70.0%)	.513

Data are presented as numbers, median (interquartile range) or mean ± standard deviations.

EQ-5D-5L = 5-level version of EuroQoL-5 dimension, EQ VAS = EuroQoL visual analogue scale, NRS = numeric rating scale, ZBPI = Zoster brief pain inventory.

* *P* < .05 by 2-tailed *t*-test.

† *P* < .05 by Mann–Whitney rank-sum test.

**Figure 3.**
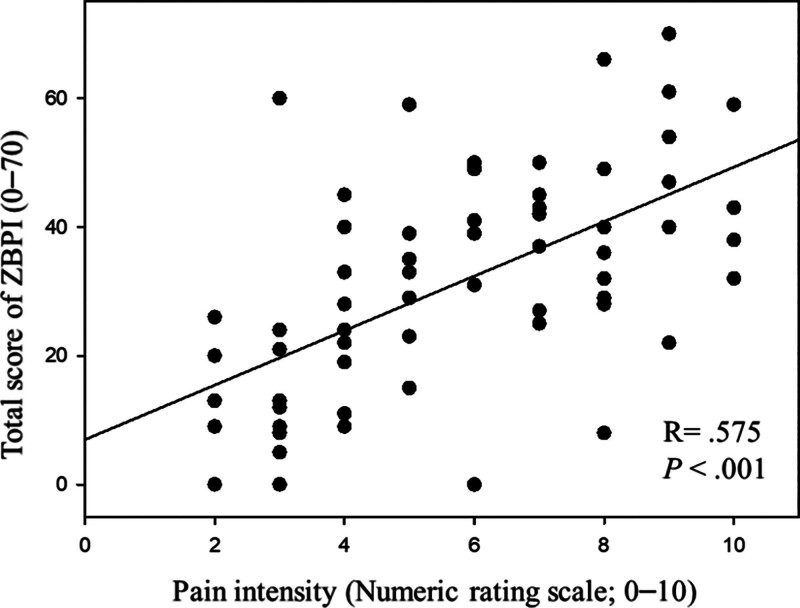
Relationship between pain intensity (NRS) and zoster pain-related interference in activities of daily living. ZBPI = zoster brief pain inventory.

The results of the univariate and multivariate linear regression analysis against ZBPI total score are shown in Table 3. In multivariate analysis, pain intensity was the only independent predictor for ZBPI total score (*R*^2^ =.381, *P* < .001). The significant correlation of between pain intensity and ZBPI total score was described in Figure 3 (*R* = .575, *P* < .001).

**Table 3:** Regression coefficient (β) and coefficient of determination (*R*^2^) against the total score of zoster brief pain inventory.

Variable	Univariate analysis				Multivariate analysis			
	β	SE	*R* ^2^	*P*	β	SE	*R* ^2^	*P*
							0.381	<.001†
Age	0.383	0.181	0.068	.039*	0.031	0.167		.853
Female sex	−7.073	4.297	0.027	.105				
Pain intensity (NRS)	4.061	0.739	0.320	<.001*	3.685	0.763		<.001†
PBF (%)	0.621	0.220	0.116	.006*	0.404	0.203		.052
SMI (kg/m^2^)	−1.592	1.904	0.011	.406				

NRS = numeric rating scale, PBF = percent body fat, SMI = skeletal muscle index.

* *P* < .05 by univariate linear regression analysis.

† *P* < .05 by multivariate linear regression analysis.

## 6. Discussions

The results of the study suggests that individuals with higher body fat may experience a greater decline in functional status compared to those with normal body composition, even when the severity of pain is similar. In particular, excessive body fat was found to negatively impact on walking ability and work-related activities in older patients suffering from PHN. Additionally, most patients with PHN reported decreased emotional well-being (indicating WHO-5 well-being index below 13 in the current study), regardless of body composition characteristics. The linkage between PHN and psychological disorders, such as depression and sleep disturbance, is well established.^[20]^ These effects can lead to a significant socioeconomic and psychological burden, increasing health care utilizations and costs in elderly PHN patients.^[21,22]^

Several previous studies have suggested that greater pain intensity is associated with increased interference in daily activities.^[4]^ The findings of the current study align with this, showing strong correlation between pain intensity and interference with ADL (described in Table 3 and Fig. 3). As a result, the authors suggests that individuals with higher pain intensity and/or abnormal body composition (increased PBF) may be at a greater risk for decreased functional status in their daily lives. These populations should receive more careful management through multimodal analgesia and support from a multidisciplinary team.

The impact of obesity on chronic pain disorders has been inconsistent across several previous studies.^[7–11]^ In those studies, BMI was traditionally used to diagnose obesity. However, the authors perceived that BMI is not the accurate measurement of body fat. Instead, a high BMI may reflect not only excessive body fat but also increased skeletal muscle mass, as it is calculated solely based on height and body weight. In contrast, body composition analysis offers a more precise evaluation of body fat, lean body (skeletal muscle) mass, bone mass, and water content, enabling more accurate diagnoses of abnormal body composition. In the current study, participants were classified into the normal adiposity and the high adiposity group based on predetermined PBF cutoff value defined by Kim et al,^[15]^ rather than BMI. Overweight was defined as having a PBF of ≥ 17 % for men and ≥ 32% for women in the Asian population. This approach, the author suggest, enhances the reliability of the study findings.

Body composition is a multidimensional concept encompassing both fat mass and skeletal muscle mass, each of which may independently influence health outcomes. While excessive adiposity is often highlighted in relation to chronic pain, skeletal muscle mass plays a critical role in maintaining physical function, particularly in older adults. From the perspective of skeletal muscle, measures such as the MFR or skeletal muscle mass index are important predictors of health outcomes. Sarcopenia, a condition characterized by a decline in skeletal muscle mass and strength, is becoming increasingly recognized, especially in the elderly population.^[23–25]^ The presence of sarcopenia may be linked to chronic musculoskeletal pain in older patients.^[26]^ Bahat et al reported that the cutoff value of skeletal muscle mass indexes to determine sarcopenia were 9.2 kg/m^2^ for men and 7.4 kg/m^2^ for women in European population.^[27]^ The Asian Working Group for Sarcopenia recommends the cutoff values of 7.0 kg/m^2^ for men and 5.7 kg/m^2^ for women when using BIA.^[28]^ Additionally, alteration in body composition involving both increased adiposity and decreased muscle mass may have compounded effects on both physical and psychological health. For instance, a lower MFR, defined as skeletal muscle mass (kg) divided by body fat mass (kg), has been reported in patients experiencing chronic pain.^[29]^ Sacropenic obesity, characterized by a low MFR, could be associated with several health issues, including chronic pain, inflammation, cognitive function, immune function, carcinogenesis and cancer survival rates.^[29–32]^ However, in the current study, no participants were diagnosed with sarcopenia, possibly due to its low prevalence in geriatric population studied. The sample size calculation was based on the primary outcome, and further research is needed to assess the impact of sarcopenia in this context.

The risk factors for PHN including severe rash and pain, old age, ophthalmic involvement, and immune suppression are well-known.^[33,34]^ In particular, Forbes et al reported that advanced age was a significant risk factor for PHN in their systemic review and meta-analysis, which the relative risk may range from 1.22 to 3.11 per 10-year increase.^[33]^ Age-related decline in cell-mediated immunity may be associated with the development of HZ and PHN.^[35]^ Meanwhile, the impact of age on the pain-related interference in ADL and QoL in PHN patients was not clearly determined. Curran et al reported that those outcomes did not vary with age.^[5]^ Similarly, the advanced age was not the statistically significant predictor for worse performance in ADL in the current study.

The current study have several limitations. First, the small sample size limits the generalizability of the findings. Additionally, cultural and demographic factors such as dietary behaviors, physical activity levels, and genetic predispositions may influence body composition, which in turn may affect PHN outcomes. Therefore, the study findings should be interpreted with consideration of different sociocultural contexts. Second, the cross-sectional design and lack of longitudinal data prevent the study from establishing cause-and-effect relationships and assessing changes over time. Third, reliance on self-reported data introduces the potential for recall bias or subjective interpretation, which may affect the results. Finally, the study focused on a specific population (elderly and Asian groups), the findings may not be applicable to other demographics or regions.

In conclusion, the results of this study suggests that individuals with higher body fat may experience reduced function in ADL and decreased QoL compared to those with normal body composition, regardless of pain severity. These finding underscore the clinical relevance of body composition on functional outcome of older patients with PHN, highlighting potential benefits of interventions aimed at improving body composition. Future studies exploring the role of sarcopenic obesity in PHN populations would be valuable to further clarify its impact on patient outcomes.

## Author contributions

**Conceptualization:** Ji-Seon Son, Aram Doo.

**Data curation:** Jeewoon Joung.

**Investigation:** Jeewoon Joung, Aram Doo.

**Methodology:** Aram Doo.

**Writing – original draft:** Ji-Seon Son.

**Writing – review & editing:** Aram Doo.
